# Mapping how information about childhood vaccination is communicated in two regions of Cameroon: What is done and where are the gaps?

**DOI:** 10.1186/s12889-015-2557-9

**Published:** 2015-12-21

**Authors:** Heather Ames, Diangha Mabel Njang, Claire Glenton, Atle Fretheim, Jessica Kaufman, Sophie Hill, Afiong Oku, Julie Cliff, Yuri Cartier, Xavier Bosch-Capblanch, Gabriel Rada, Artur Muloliwa, Angela Oyo-Ita, Simon Lewin

**Affiliations:** Global Health Unit, Norwegian Knowledge Centre for the Health Services, Boks 7004, St Olavs plass, N/0130, Oslo, Norway; Department of Anthropology, University of Yaoundé 1, BP 337 Yaoundé, Central Province, Cameroon Africa; Institute of Health and Society, University of Oslo, P.O box 1130 Blindern 0318, Oslo, Norway; Centre for Health Communication and Participation, C/o Department of Human Biosciences, College of Science, Health and Engineering, La Trobe University, Melbourne campus, 3086 VIC Australia; University of Calabar, Nigeria, P.M.B 1115 Calabar Municipal, Cross River State Nigeria; Faculdade de Medicina, Universidade Eduardo Mondlane, Maputo, Mozambique Africa; International Union for Health Promotion and Education, 42 Blvd. de la Libération, 95203 St, Denis, Cedex, France; Swiss Tropical and Public Health Institute, Socinstrasse 57, 4051 Basel, Switzerland; University of Basel, Petersplatz 1, 4003 Basel, Switzerland; Evidence-based Healthcare Program, Pontificia Universidad Católica de Chile, Avda. Libertador Bernardo O’Higgins 340, Santiago, Chile; Direcção Provincial de Saúde de Nampula, Departamento de Saúde, Av. Samora Machel n° 1016 R/C, C.P. N° 14, Nampula-Moçambique, Africa; Health Systems Research Unit, South African Medical Research Council, Francie van Zijl Drive, Parowvallei, Cape Town, PO Box 19070, 7505 Tygerberg South Africa

**Keywords:** Childhood vaccination, Immunization, Communication, Low- and middle-income country, Cameroon, Intervention, Consumer, Taxonomy, Parents, Caregivers, Demand generation, Vaccine hesitancy

## Abstract

**Background:**

The ‘Communicate to vaccinate’ (COMMVAC) project builds research evidence for improving communication with parents and communities about childhood vaccinations in low- and middle-income countries. Understanding and mapping the range of vaccination communication strategies used in different settings is an important component of this work. In this part of the COMMVAC project, our objectives were: (1) to identify the vaccination communication interventions used in two regions of Cameroon; (2) to apply the COMMVAC taxonomy, a global taxonomy of vaccination communication interventions, to these communication interventions to help us classify these interventions, including their purposes and target audiences; and identify whether gaps in purpose or target audiences exist; (3) to assess the COMMVAC taxonomy as a research tool for data collection and analysis.

**Methods:**

We used the following qualitative methods to identify communication strategies in the Central and North West Regions of Cameroon in the first half of 2014: interviews with program managers, non-governmental organizations, vaccinators, parents and community members; observations and informal conversations during routine immunization clinics and three rounds of the National Polio Immunization Campaign; and document analysis of reports and mass media communications about vaccination. A survey of parents and caregivers was also done. We organised the strategies using the COMMVAC taxonomy and produced a map of Cameroon-specific interventions, which we presented to local stakeholders for feedback.

**Results:**

Our map of the interventions used in Cameroon suggests that most childhood vaccination communication interventions focus on national campaigns against polio rather than routine immunisation. The map also indicates that most communication interventions target communities more broadly, rather than parents, and that very few interventions target health workers. The majority of the communication interventions aimed to *inform or educate* or *remind or recall* members of the community about vaccination. The COMMVAC taxonomy provided a useful framework for quickly and simply mapping existing vaccination communication strategies.

**Conclusions:**

By identifying the interventions used in Cameroon and developing an intervention map, we allowed stakeholders to see where they were concentrating their communication efforts and where gaps exist, allowing them to reflect on whether changes are needed to the communication strategies they are using.

**Electronic supplementary material:**

The online version of this article (doi:10.1186/s12889-015-2557-9) contains supplementary material, which is available to authorized users.

## Background

Vaccination has been described as one of the greatest public health achievements of the 20th century [1], and is widely seen as a worthwhile and cost-effective public health measure. However nearly 22 million infants, mainly in low- and middle-income countries, did not receive the full series of basic immunisations in 2012 [2–4], contributing to 1.5 million preventable deaths [5].

Low vaccination uptake can be attributed in part to vaccine hesitancy as well as other barriers such as access, finances and lack of infrastructure. Vaccine hesitancy is defined by the World Health Organization as a ‘delay in acceptance or refusal of vaccines despite the availability of vaccine services. Vaccine hesitancy is complex and context specific, varying across time, place and vaccines. It is influenced by factors such as complacency, convenience and confidence.’ [6]. Factors that may determine an individual’s vaccine hesitancy have been categorised into three domains: contextual influences, including socio-cultural and health systems factors; individual and group influences, including those arising from personal perceptions of a vaccine; and vaccine or vaccination-specific issues, including individual assessments of risks and benefits and the effects of the mode of administration [7].

A number of studies and reviews have explored the reasons for vaccine hesitancy and the non-vaccination of children [6, 8, 9]. Overall, they highlight that vaccination decision making is a complex process, influenced by many factors. An important barrier for individuals in many settings is a lack of appropriate information, leading to doubts about the trade-offs between the benefits and harms of vaccination and to fears about side effects or other implications [10–14]. People may lack knowledge about how vaccinations ‘work’ and about the diseases that vaccines prevent [10, 12, 15]. When communication about vaccination is poor or inadequate it can negatively affect vaccination rates and undermine vaccine acceptance [6]. Therefore, improving communication about vaccination is a key factor in improving vaccination outcomes [16, 17] and achieving the wider goal of knowledgeable parents and communities – important contributors to improving child health in many settings [18–20].

Within the ‘Communicate to vaccinate’ (COMMVAC) project – an initiative to build research evidence for improving communication with parents, caregivers and communities about childhood vaccinations in low- and middle-income countries – communication is defined as a purposeful, structured, repeatable and adaptable approach to inform and influence individual and community decisions in relation to personal and public health participation, disease prevention and promotion, policy making, service improvement and research [21, 22]. A single communication intervention, such as an information leaflet for parents, may be used on its own or with other interventions as part of a larger communication strategy [21, 22]. Communication about vaccination may target one or more groups for a specific purpose. For example, a communication intervention may target parents in order to provide information to inform their decisions on vaccinating their children.

To improve communication regarding childhood vaccination it is important to know what interventions are being used, where, targeting whom and for what purpose [23]; what communication interventions are effective [24–26]; and how people want to be communicated with. For many low- and middle-income countries, we have limited knowledge of which communication interventions are used around childhood vaccination and for what purpose, as reports and studies often do not provide sufficient detail and interventions are often not clearly identified or organized. This limits our understanding of the range of interventions being used as well as how these are used, and therefore hinders evidence-informed decision making regarding vaccination communication interventions and strategies [23].

In response to the lack of a comprehensive approach to identifying and organising the broad range of communication interventions used to improve childhood vaccination uptake globally, the COMMVAC project has developed a classification system, or taxonomy [27]. The studies used to develop this taxonomy of vaccination communication interventions were drawn largely from the indexed literature and were therefore weighted towards interventions implemented in high-income settings [23, 26]. To address this, the COMMVAC project is now using the COMMVAC taxonomy to create a local map of the vaccination communication interventions used in several low and middle-income country settings: Central and North West Regions in Cameroon, Cross River and Bauchi States in Nigeria and Nampula Province in Mozambique. These settings were chosen to provide variation in vaccination coverage, vaccination delivery, and health systems organization.

Cameroon, one of the settings for this work, follows WHO recommendations for routine immunizations for children [28]. Based on the 2011 demographic health survey, 53 % of Cameroonian children are fully vaccinated, with a range of 31-83 % across the ten regions of the country [29]. Five percent of children receive no vaccinations at all [29]. In its recent 2011-2015 multi-year plan, the Cameroonian vaccination program identified a lack of focus on routine vaccination communication. The plan cited insufficient implementation of communication interventions; low levels of ‘passion’ of health district supervisors for communication activities; low levels of financing; insufficient involvement of stakeholders such as opinion leaders, traditional leaders, and religious authorities; and a lack of training of focal communication persons [30]. This lack of focus on routine vaccination communication has been compounded by a polio epidemic which diverted attention away from the routine vaccination program towards running a series of national immunization days against polio in 2014-15 [31]. In this paper, we describe the research conducted in Cameroon in 2014 to map the childhood vaccination communication interventions being used in the North West and Central Regions of the country.

### The study objectives

Our first objective was to identify the vaccination communication interventions used in two regions of Cameroon for vaccination campaigns and for routine childhood vaccination.

Our second objective was to apply the COMMVAC taxonomy to the interventions identified in these Cameroonian contexts to help us classify which communication interventions were being used; for what purpose; and for which target audiences; and to identify any gaps in purpose or target audiences.

Our third objective was to assess the COMMVAC taxonomy as a research tool for data collection and analysis in the field of vaccination communication.

## Methods

### The COMMVAC Taxonomy

The COMMVAC taxonomy was developed through a rigorous process of literature review and consultation with expert groups, and also draws on earlier taxonomies developed for communication interventions in general [20]. The taxonomy includes interventions that influence interactions between health care providers and consumers as well as interventions that involve communication with, and participation of, parents, informal caregivers and community members. The taxonomy does not include health systems interventions focused on the funding of vaccination programmes or how vaccination itself is delivered (for example, making vaccination services more accessible, improving the training of health care providers in vaccination delivery or providing incentives to consumers or providers to improve vaccination uptake) [23].

**Fig. 1:**
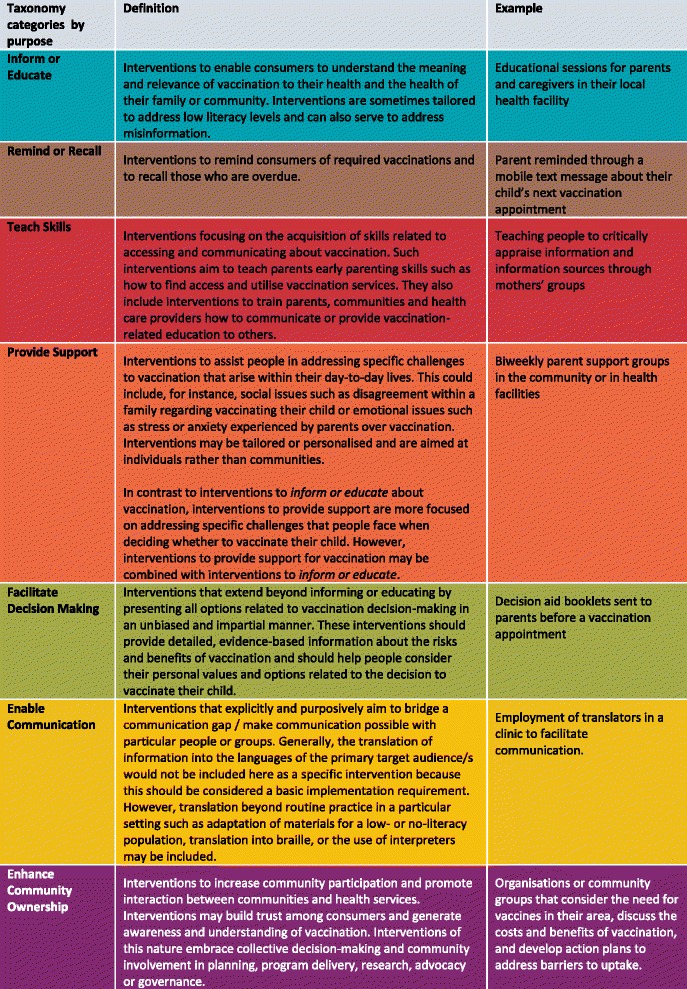
The ‘communicate to vaccinate’ taxonomy – purposes, definitions and examples^a^

The taxonomy organises communication interventions into seven categories based on their intended purpose for three target groups: parents, communities, and health care providers (Figure 1). We define purpose as the intended goal of the communication intervention for the target group. By clarifying the key purposes and features of interventions, the taxonomy can aid implementation and evaluation and can be used to identify areas where interventions are being used and where gaps exist [27].

### Setting

We conducted the current study in the Central and North-West regions of Cameroon. These two regions were selected purposively to ensure coverage of both French and English language areas and rural and urban settings. Yaoundé, the country’s capital and located in the Central region, provided the urban setting, with research taking place in three urban health districts. 60 % of children in Yaoundé are completely vaccinated [29]. The North-West region provided the rural setting for the study, with all research activities taking place in one rural health district. 83 % of children in this region are fully vaccinated [29]. However, there are pockets of low vaccination completion in the hard-to-reach rural areas of the region.

During fieldwork, there were monthly National Immunization Campaigns against Polio in response to the discovery of indigenous polio in Cameroon after several years without any cases [32]. The Cameroonian Extended Program of Immunization (EPI) also introduced the rotavirus vaccine to the routine childhood vaccination program in April 2014 [33].

Settings for data collection in both regions included health facilities, district health offices, schools, churches, and communities. We studied communication interventions for both routine and campaign vaccination. By routine vaccination, we mean vaccinations delivered as part of the extended program of immunization (EPI) following the vaccination calendar at fixed or outreach sites. By campaigns, we mean any vaccination activity that happens outside of the routine structure and seeks to reduce the transmission of particular, selected vaccine preventable diseases in an age group (of children) that is expanded for the duration of the campaign [34].

### Data collection and analysis

Between January and May 2014, we identified communication interventions related to childhood vaccination in Cameroon. The principle investigator (HA) and research assistant (DMN) both speak French and English, the two official languages of Cameroon. We conducted most of the interviews and observations in the Central region of Cameroon in French, while most of the interviews and observations in the North-West region were conducted in English or Pidgin English (a language spoken in some parts of Cameroon). The research assistant (DMN) is from the North West Region, speaks fluent Pidgin English, led some interviews and acted as a translator.

We used purposive sampling to select the health facilities included in the study. As with the selection of regions, sampling aimed to ensure urban and rural settings covering both English and French language areas with varying vaccination coverage. We also used purposive sampling for most participants apart from parents in order to ensure a range of participants from different levels of the health services and with different roles within vaccination communication. Convenience sampling was used to select the parents to be interviewed, and was usually dependant on how much time they had available and if they were interested in participating while attending a vaccination session at a health facility.

All interview participants signed an informed consent form or agreed orally to participate in the interview once the consent form had been explained. Access to observe vaccination delivery was granted by the person in charge of the health facility. Permission to observe the vaccination campaigns was given by the local person in charge of the campaign.

For each identified intervention, we collected as much information as possible about the target group/s, frequency, planning, content and timing.

**Fig. 2:**
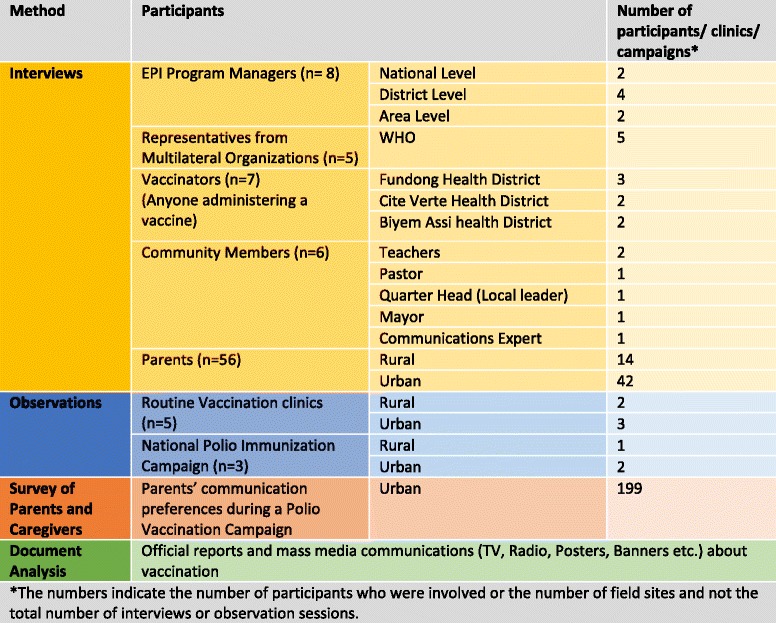
Overview of data collection methods and participants

Figure 2 presents an overview of the data collection methods.

### Semi-structured interviews

We (HA assisted by DMN) conducted semi-structured interviews with all relevant stakeholders involved in vaccination activities, namely: vaccinators, program managers, representatives from multilateral organizations, community members, and parents. We conducted the interviews in vaccination clinics, offices, churches and schools to explore participants’ experiences with vaccination communication interventions. We asked interview participants to discuss communication interventions that they had experienced or were aware of in their setting and their perceptions of these interventions, and to identify documents used for communication about vaccination (see below).

### Participant observation and informal conversations

We carried out participant observation and informal conversations during routine immunization activities and in three rounds of the National Polio Immunization Campaign. This was done to complement what was said in interviews with what was taking place in vaccination sessions and campaigns. Observations were done in vaccination clinics and communities during vaccination campaigns. During these observations, we also conducted informal conversations with vaccinators, social mobilisers (community members trained as lay health workers to deliver health promotion messages) and parents. The focus of observations was on communication in the vaccination setting, the interactions between the various groups involved and the content of the information given about vaccination.

### Document and media analysis

We asked participants about documents they used to plan or deliver vaccination communication interventions. During fieldwork, we also collected media articles and stories about vaccination and any vaccination related items, such as vaccination cards, posters or banners. After the completion of fieldwork, we carried out an internet search of relevant country websites to locate documentation describing communication for vaccination in Cameroon. We reviewed the documents that we managed to identify and locate in order to identify any additional interventions that were not observed or mentioned in the interviews. The document analysis allowed us to complement what we were observing and the interview data with what was being described in plans, reports and media stories.

### Survey of parents and caregivers

As mentioned above, we accompanied different vaccination teams during three polio immunization campaigns in February, March and April 2014. During the April campaign, we also carried out a survey composed of eight questions addressing how caregivers had heard about the vaccination campaign and the new rotavirus vaccine and what their preferred communication channel would be to receive information about vaccination. The survey was partially developed based on a discussion with the EPI office about what kind of information would be useful to them. The survey was done opportunistically to make use of the interaction with caregivers as part of our observation of a campaign. The purpose of the survey was to identify any interventions that were missed by other data collection methods and to use this information to check the draft map of vaccination communication interventions for completeness. During the course of the two days, we administered the survey at each household where the vaccination team administered a polio vaccine. We carried out the survey verbally with the caregiver and recorded the answers on a standard survey form after the vaccination team had spoken with the household. (Additional file 1).

### Data analysis

We went through all of the collected data and extracted all information about the communication interventions being used. We then used the COMMVAC taxonomy of vaccination communication strategies [27] to organise the collected data into a descriptive map showing which strategies were being delivered to whom, how, and for what purpose. This was an ongoing, iterative process and early drafts of the Cameroonian map were presented to participants as the study progressed to validate the interventions collected and suggest any that were missing. We discussed any uncertainty in how to classify interventions within the COMMVAC team.

At the end of fieldwork, we presented the preliminary Cameroonian map at a meeting of the Extended Program of Immunization (EPI) Cameroon, including members of the WHO country office, for feedback on its completeness.

Ethical clearance was granted in Cameroon by La Comité National d’Ethique de la Recherche pour la Santé Humaine (CNERSH).

## Results and discussion

### The map of vaccination communication interventions used in Cameroon organized by purpose

**Fig. 3:**
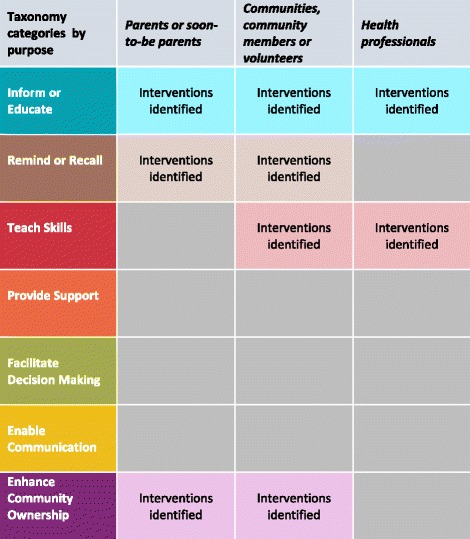
Overview of the Cameroonian map of vaccination communication interventions

Figure 3 provides an overview of the Cameroonian map of vaccination communication interventions and indicates the COMMVAC taxonomy categories for which interventions were identified. The complete map of vaccination communication interventions in Cameroon, organised using the COMMVAC taxonomy, is available in Additional file 2. A document describing in detail all of the individual interventions identified is available from the authors on request. For specific definitions of each taxonomy purpose, refer to Figure 1.

#### Interventions to inform or educate

By far the most common specific purpose of vaccination communication interventions used in Cameroon was to *inform or educate*. The majority of these were geared towards communities. The focus was on informing people about the dates, locations, target ages and reasons for the upcoming campaigns. In both urban and rural settings, this was done through mass media, social mobilisers (community members trained to deliver health promotion) and announcements at community focal points such as churches, community-based organisations, schools and the houses of quarter heads (neighbourhood leaders). Town criers or announcers with loud speakers were also used. In addition, the Ministry of Health has started to use SMS-technology to inform the general population about upcoming campaigns and the introduction of new vaccines to the EPI program, but this technology is not yet used to inform people about routine vaccination. Recently, a famous football player was used in television advertisements to inform and educate about vaccination and the first lady of Cameroon was the ‘godmother of vaccination’ for the introduction of the new rotavirus vaccine, helping to inform the public about the new vaccine.

When parents were targeted, they were approached at the clinic or at their home during door-to-door campaigns. However, the door-to-door campaigns can also be seen to target communities as every house was approached, not just those with children. Houses with no children were given minimal information and the team moved on quickly to the next house. At clinics, parents were usually given a health talk before the vaccination session. These health talks typically included information about the vaccinations their child would receive that day and when to return for their next vaccination appointment. Sometimes the vaccinators would sing songs about vaccination during health talks. Another strategy targeting parents was the vaccination card received and filled in at their child’s first routine vaccination. Although the amount of information in the vaccination cards is limited, parents noted that they saw them as an important source of information. Furthermore, a maternal and child health handbook that will contain more health information was being pretested in one of the health districts in the study. This 74-page handbook will provide caregivers with more in - depth maternal and child health information about topics such as vaccination, nutrition, breast-feeding and injury prevention using both text and pictorial instructions.

Health workers were rarely the target of a communication strategy to *inform or educate* about vaccination. If targeted, this was during training for campaigns or for the introduction of a new vaccine where communication training forms a small part of the larger technical training. For example, during the introduction of the rotavirus vaccine, vaccinators were taught how to inform parents about the new vaccine through role-plays. This training may build on any pre-service training they received.

#### Interventions to remind or recall

We found a large number of interventions with the specific purpose of *reminding or recalling* people about vaccination appointments and campaigns. These interventions may be specifically tailored to remind an individual to return for a vaccination on a particular date, or they may be more general. In Cameroon, we found a large overlap in the communication interventions that aim to *inform or educate* and to *remind or recall*, because most vaccination messages included a general reminder to come for your next vaccination or to follow the vaccination calendar.

The majority of the interventions to *remind or recall* targeted communities and focused primarily on vaccination campaigns. The most commonly used channels were mass media, and announcements in churches, schools, community-based organizations, community focal points and via town criers. Social mobilisers also played an important role in reminding the community about an upcoming campaign by going door- to-door in their neighbourhoods. There has been an increase in the use of SMS to remind the general population about the dates and target age of vaccination campaigns in Cameroon. However, SMS reminders for individual appointments were not used.

In both urban and rural settings, parents were targeted through health talks and during vaccination appointments where they were told by health workers when to bring their child back for the next vaccination. This was also written in their vaccination card. In the rural setting, the health centre sent out social mobilisers to trace children who had missed a vaccination appointment and to remind families to bring the child to the health centre.

#### Interventions to teach skills

Very few of the interventions we identified specifically aimed to teach people skills about communicating information about vaccination and none of the interventions we identified taught people how to access information about vaccination. All of the examples that we found in this category in Cameroon involved training people in how to provide or communicate information to others. For instance, church pastors received training from the focal communication person for a district on how to better communicate about vaccination to their congregation. Most of these interventions were linked with community advocacy meetings. Advocacy strategies were used when communities had been identified as having some vaccine hesitancy, e.g. a church or group who would not vaccinate, or low vaccination coverage in an area. They covered both routine vaccination and campaigns. These often took place with members of churches or community-based organizations. Social mobilisers, or those managing them, met with leaders of these groups to discuss the importance of vaccination and communicating this to their group members.

Health workers were taught how to communicate with parents during training sessions. These training sessions could happen during their pre-service training, before each campaign, and/ or before the introduction of a new vaccine. This was done through role-play and discussion. They also received some training on how to deliver the health talks that they give at clinics before vaccination sessions.

#### Interventions to provide support

We recorded no examples of interventions with the specific purpose to *provide support*.

#### Interventions to facilitate decision-making

We recorded no examples of interventions with the specific purpose to *facilitate decision making*.

#### Interventions to enable communication

We observed no specific interventions aimed at *enabling communication* about vaccination. However, in the health facilities, health care providers generally spoke the local language(s) of the community where they were working. The health talks given by the vaccinator as well as the vaccination songs were in a language understood by the majority of those visiting the clinic. The print materials used to support vaccination, such as posters and vaccination cards, were in French or English depending on the region of the country. The only time participants referred to a language issue was if an area received promotional materials in the wrong language.

#### Interventions to enhance community ownership

We observed a few interventions that aimed to *enhance community ownership* around childhood vaccination. In Cameroon, the EPI program set out to actively build partnerships with local organisations for the promotion of vaccination. This was usually done through social mobilisation, with most of the focus being placed on campaigns. The most common strategy was for social mobilisers to liaise with community members and community organisations to involve them in the promotion of vaccination in the community. The most visible forms of community involvement and ownership were observed during the vaccination campaigns where community members would volunteer to lead the vaccination team through their neighbourhood to show them where children lived.

Parents were encouraged by health workers to talk about vaccination in their communities. Local opinion leaders, including quarter heads, imams, pastors and teachers, were also used to promote vaccination locally.

#### Communication interventions proposed by participants

**Fig. 4:**
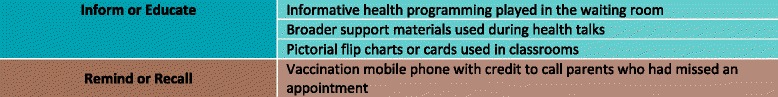
Communication interventions proposed by participants

During the study, participants, including health workers and teachers, suggested communication interventions that were not currently in practice but that they felt could be developed (See Fig. 4). The following strategies fell into the category of interventions to *inform or educate*. During fieldwork, we observed that most waiting rooms had a television that played popular programmes such as soap operas. Vaccinators thought that these could easily be used to show informative health programmes to mothers. Vaccinators also wanted an increased range of support materials that they could use during health talks to engage their audiences. These could take the form of posters, pictorial teaching tools or pamphlets that could be given to mothers to take home. School teachers wanted pictorial flip charts or cards that they could use to inform or educate their class about the vaccinations they were receiving during the campaign and vaccination in general.

Another suggestion proposed by health workers fell into the *remind or recall* category. They said that although the phone numbers of parents were recorded in their ledger they were unable to call and follow up when parents missed an appointment. If they wanted to call, they had to use their private phones at their own cost. They recommended that each vaccination room be given a mobile phone with credit to follow-up with parents who had missed a vaccination appointment.

### Organization of the Extended Program of Immunization

We found that the communication strategies in Cameroon tended to use a top-down approach. The communication interventions used in campaigns were for the most part developed at the national level by the EPI program or by international organizations such as the WHO or UNICEF. These materials and interventions were then presented in national media or sent out to the regional offices, from where they were passed onto the district health officers, who then distributed them to their local health areas. These materials were rarely, from our observations, adapted to local contexts. As the print materials came in standard form from the national level, they did not include local information and sometimes came in the wrong language. Often materials arrived at the last minute or arrived after the start of the campaign, leaving no time for adaptation, or did not arrive at all. Communication was also top-down in the sense that the interventions focused on providers delivering information to people.

### Using the COMMVAC taxonomy as a research tool to collect and analyse data

We found that the COMMVAC taxonomy allowed us to create a detailed and structured overview of the strategies that we identified in the data. When presenting the taxonomy framework and Cameroonian map to study participants, their feedback was that these were easy to understand and gave them a simple visual overview of how communication strategies were being used in their setting. However, the majority of participants who were asked about the map did not distinguish between interventions designed to *inform or educate* and interventions designed to *remind or recall* because the vast majority of messages about vaccination in Cameroon end with a reminder to attend your next vaccination appointment or to join in the next campaign. During the final stage of feedback collection, in a meeting with the EPI program to present the taxonomy, one further strategy, la boîte aux images or ‘picture box’, was identified. This is a series of pictures that are presented and explained during a talk [35, 36].

Another challenge of the mapping process was how to classify communication strategies that addressed multiple purposes and multiple target groups. For this paper, we have classified interventions by main target group and or purpose. However, a few of the interventions could be placed under multiple categories.

### Discussion

Our study shows that the focus in Cameroon at the time of fieldwork was on communicating about vaccination campaigns. The majority of vaccination communication interventions aimed to *inform or educate* or to *remind or recall*. The main target group for these interventions were the community, followed by parents and then health workers.

This focus on campaigns, as opposed to routine immunization, is related to the international polio eradication initiative and the polio outbreak in 2013-14, which led to multiple national immunization campaigns. This focus also seems to have led to a shift from communicating directly with parents to targeting a broader audience: the community. The frequent use of mass media (for example radio, TV, newspapers, SMS etc) as a communication channel can also be seen as a consequence of the focus on campaigns and on the community. Interventions targeting parents, on the other hand, were mostly delivered at health centres. Very few interventions focused on health workers; when they were targeted, it was during training days for campaigns or for the introduction of a new vaccine.

The top - down structure of the extended program of immunization may have limited the extent to which communication materials could be adapted to local settings. It has been suggested that for vaccination communication programs to be successful they should be developed taking local needs and knowledge into consideration in order to make them relevant to local contexts [37]. However, to develop more local communication materials and strategies the capacity of health workers and district EPI programs would need to be strengthened and this would require considerable resources.

We categorised many of the interventions we found as belonging to the *inform or educate* category of the taxonomy. However, the aim of this information appeared primarily to be to get parents to bring their children for vaccination and to complete the vaccination calendar on time as opposed to giving parents and community members an understanding of what each vaccination was for and why people should be bringing their children to vaccination clinics. This may be an important gap in the way in which information is being provided.

When provided with vaccination information in health facilities, many mothers did not actively engage vaccinators even when asked if they had questions. This may indicate a general lack of patient participation or shared decision-making in health care choices in this setting. Some mothers also mentioned that they did not know that they could ask questions during the vaccination visit and so had not thought about what further information they would like to know. This lack of information and or shared decision-making could possibly lead to decreased trust in the vaccination program if a vaccination rumour were to appear. If rumours do appear, parents may not have the information necessary to understand why vaccination is important and why the rumour could be false, as demonstrated by the Tetanus Toxoid scare in the 1990s in Cameroon [38]. A forthcoming paper will explore parents and caregivers’ views on the vaccination communication they received.

There are advantages and disadvantages to the approaches to vaccination communication that we mapped in Cameroon. By focusing on mass campaigns, targeting the community and using mass media, the health services were able to reach large numbers of people in a short space of time. The centralised development of the communication interventions also made the process simpler and possibly cheaper than if individual strategies had been developed for each area. However, mass media communication interventions tend to be uni-directional and do not generally allow for discussion and questioning from those receiving the information. In addition, the focus on the national vaccination campaigns against polio for children from 0-5 years appeared to lead to less emphasis on communication about routine vaccinations for children from 0-11 months.

### Using the COMMVAC taxonomy

The COMMVAC taxonomy allowed us to create order in the complexity and range of communication strategies emerging from the fieldwork, and enabled us to examine which vaccination communication interventions are used and where gaps in communication interventions exist. In addition, the taxonomy framework was a useful tool during interviews as we were able to present the incomplete map to participants and ask for feedback, allowing us to check the validity and completeness of the findings.

The completed taxonomy also allows those working with vaccination communication to identify gaps in their own communication strategies as it can highlight relevant target audiences or purposes that they may have missed. By grouping the interventions by purpose in the map, program managers are able to make sure that the interventions they are using address key aspects of vaccine hesitancy in their local context, for example linked to lack of information or misinformation. They can then map these gaps and develop interventions to address them. For instance, the taxonomy could be used to identify specific, tailored communication interventions for high priority vaccination hesitancy groups, such as parents of children who have not been vaccinated at all.

### Strengths and limitations of the study

The main strength of the study was the iterative process that we adopted when populating the taxonomy, through first identifying interventions, then collecting feedback on preliminary versions of our intervention map from participants, which led to the identification of new interventions. This iterative process also gave participants the opportunity to give feedback on the usefulness and layout of the taxonomy itself. Another strength was the collection of data in four health districts, in two regions of Cameroon. These districts represented both urban and rural settings, different levels of vaccination coverage, and different cultural/ethnic/language groups. A potential limitation of the study is that it was conducted during a polio epidemic where there was an increased focus on campaign activities. This could have influenced the data we collected, particularly the extent to which campaigns were the focus of vaccination communication activities and received priority over routine vaccination activities. Finally, as many of the communication interventions used are not formally documented in reports and programme plans, we relied on participants’ reports and our observations in the field to detail what was done. This approach may be more susceptible to recall bias.

## Conclusions

The map of vaccination communication interventions (Additional file 2 and Fig. 3) provides an overview of the activities that were being undertaken in two regions of Cameroon at the time of the study. It also identifies areas where efforts could be made to consider how caregivers’ communication needs could be better addressed. For instance, more attention could be paid to communication about routine vaccination. To build communication interventions that focus on the needs of parents and caregivers, it is important to understand what they want from the vaccination communication they receive. This will be the focus of a forthcoming paper exploring Cameroonian parents’ communication preferences as well as a qualitative evidence synthesis on parents' and caregivers’ views about early childhood vaccination information.

This study suggests that the COMMVAC taxonomy has a range of applications within childhood vaccination programmes at national and sub-national levels. Firstly, it can assist programme managers in mapping the range of communication strategies they are using in a way that identifies the key purposes of each strategy (for example, to enhance community ownership of childhood vaccination programmes). This novel way of organising strategies may help programme managers to ensure that the communication strategies that they are using address specific determinants of vaccine hesitancy. Secondly, it can help programme managers and researchers standardise the description of strategies within and across countries, making it easier to compare the approaches used in different settings. Finally, the taxonomy can help programme managers to identify key types of communication strategies (e.g. teaching skills, providing support) that they are not using widely and that might be useful if scaled up. In forthcoming papers, we will explore the similarities and differences in the range of interventions being used across the COMMVAC project sites in Mozambique, Nigeria and Cameroon.

## Additional files

Additional file 1:
**Questions included in the survey of parents and caregivers.** (PDF 171 kb) Additional file 2:
**Map of vaccination communication interventions in Cameron.** (PDF 450 kb)

## Abbreviations

COMMVAC: The Communicate to Vaccinate Project
EPI: Expanded Program on Immunization
UNICEF: United Nations Children’s Fund
WHO: World Health Organization
