# Supplementary material for: Mapping how information about childhood vaccination is communicated in two regions of Cameroon: What is done and where are the gaps?
Source: BMC Public Health. 2015 Dec 21;15:1264. doi: 10.1186/s12889-015-2557-9 (PMC4687068; doi:10.1186/s12889-015-2557-9)
Supplement: Additional file 1: — Questions included in the survey of parents and caregivers. (PDF 171 kb) [file 12889_2015_2557_MOESM1_ESM.pdf]

Supplementary document 1

Questions included in the survey of parents and caregivers:

1) Do you know which disease we are vaccinating against today?
2) Have you heard about the new cases of polio in Cameroon?
3) Do you know that there was going to be a campaign this weekend?
4) If yes, how did you hear about the campaign?
5) What is the easiest way to inform you about the vaccination campaigns?
6) Have you heard that there is a new vaccine for babies 0-11 months?
7) Do you know what the vaccine is given for?
8) If yes, how did you hear about the new vaccine?

The survey was partially developed based on a discussion with the EPI office about what kind of information would be useful for them to have.
