# Supplementary material for: Mapping how information about childhood vaccination is communicated in two regions of Cameroon: What is done and where are the gaps?
Source: BMC Public Health. 2015 Dec 21;15:1264. doi: 10.1186/s12889-015-2557-9 (PMC4687068; doi:10.1186/s12889-015-2557-9)
Supplement: Additional file 2: — Map of vaccination communication interventions in Cameron. (PDF 450 kb) [file 12889_2015_2557_MOESM2_ESM.pdf]

Map of vaccination communication interventions in Cameron

Red text = Interventions included in campaigns only

Blue text = Interventions included in routine vaccination only

Purple text = Interventions included in both campaigns and routine

Green text = Interventions that participants suggested could be included

| | *Parents or soon-to-be parents* | *Communities, community members or volunteers* | *Health professionals* |
|---|---|---|---|
| **Inform or Educate** | **One on one interactions** | **One on one interactions** | **One on one interactions** |
| | | Social mobilizers who go door to door before the campaign to inform and map children<br><br>Social mobilizers who go out with the vaccination team during campaigns<br><br>Communicating with the neighbourhood chief<br><br>Outreach activities for EPI vaccinations | |
| | **Group interactions** | **Group interactions** | **Group interactions** |
| | Health talks before vaccination sessions<br><br>Singing the vaccination song at the rural clinic | Announcements in church<br><br>Announcements in Community Based Organizations (CBOs)<br><br>Talking to kids at schools before or during campaigns<br><br>Explanations to teachers and administrators at schools about the campaign<br><br>Vaccination teams visiting churches and schools during campaigns | One day vaccinator training for campaigns<br><br>One day training for vaccinators about the new vaccine |
| | **Mail** | **Mail** | **Mail** |
| | | | |
| | **Phone-based** | **Phone-based** | **Phone-based** |
| | | SMS sent by the ministry of health (only campaigns unless launching a new vaccine for EPI) | |
| | **Device or tool** | **Device or tool** | **Device or tool** |
| | The Vaccination Card<br><br>The Child Health Book (Carnet de Santé) | The vests worn by the vaccinators with information printed on them | |

| | | | |
|---|---|---|---|
| | **Audio visual / performance** | **Audio visual / performance** | **Audio visual / performance** |
| | | Health programming that can be shown on the TV in the primary health clinic waiting room<br><br>The organization of live performances at community hot points | |
| | **Printed material** | **Printed material** | **Printed material** |
| | The vaccination calendar poster hanging in the vaccination clinic<br><br>Small pamphlets to send home with mothers containing the information from the Health Talk<br><br>A flip chart with vaccination information and illustrations to use during health talks<br><br>La boite aux images | Posters in waiting rooms or in the community i.e. at churches or schools (however these have very different messages for campaigns and routine)<br><br>Letters to schools, CBOs and churches from DMO informing about the campaign<br><br>Information sent to schools for local health centres to inform about an upcoming campaign<br><br>Posters, banners and pamphlets (mostly campaigns unless launching a new vaccine for EPI)<br><br>An educational flip chart to use in classrooms to teach children about vaccination<br><br>Small pamphlets or coupons about a vaccination campaign to send home with children from school<br><br>The media pack given to journalists before the launch of the rota virus vaccine<br><br>Announcement board at the Quarter Head or community leader's house | The handbook of norms and standards produced by the Ministry of Health |
| | **Web-based** | **Web-based** | **Web-based** |
| | | | |

| | | | |
|---|---|---|---|
| | | **Media campaign** | |
| | | Town crier<br><br>Caravan with radio loudspeaker<br><br>Radio Announcements<br><br>Interactive Radio Programming<br><br>TV Spots<br><br>Interviews on TV or TV Health Programming<br><br>TV News casts<br><br>Reports in News Papers | |
| | | **Community event** | |
| | | Gathering at the quarter head's house or a church to be vaccinated<br><br>Giving vaccinations at community events such as the Women's Day Parade<br><br>Advocacy meeting with church goers or members of a CBO<br><br>Mobilization meetings with CBOs, Health Committees or Community Leaders | |
| | | **Celebrity spokespeople** | |
| | | The first lady launched the new rotavirus campaign and is known as the godmother of vaccination<br><br>A football player was used in adds to promote vaccination | |
| **Remind or Recall** | **One on one interactions**<br>Social Mobilizers who are sent into the community to follow up drop outs<br><br>Reminder of the next vaccination at the clinic<br><br>**Group Interactions**<br>Health Talks at routine vaccinations to remind about the next appointment<br><br>Health talks at routine vaccinations that remind about an upcoming campaign | **One on one interactions**<br>Social mobilizers going door to door to remind about the campaign and map children<br><br>Interactions with community leaders to remind them of an upcoming campaign and ask them to remind their communities<br><br>**Group Interactions**<br>Announcements in church<br><br>Announcements in CBOs | **One on one interactions** |

| | | | |
|---|---|---|---|
| | | Announcements in Schools | |
| | **Mail** | **Mail** | **Mail** |
| | | | |
| | **Phone-based**<br>A phone with credit to call parents who have not come for their appointments | **Phone-based**<br>SMS reminder about upcoming campaign<br><br>**Media Campaign***<br>Town crier<br><br>Radio Announcements<br><br>TV Spots<br><br>TV News casts<br><br>Reports in News Papers<br>*These media sources are included here as well as under *inform and educate* as when they are repeated they become a form of remind and recall. | **Phone-based** |
| | **Device or tool**<br>The Vaccination Card<br><br>The Child Health Book (Carnet de Santé)<br><br>**Printed Material**<br>Poster of the vaccination calendar at the EPI Clinic | **Device or tool**<br>A statue promoting vaccination that was located in the middle of the busy traffic circle<br><br>**Printed material** | **Device or tool** |
| | **Training in how to communicate/ provide education to others** | **Training in how to communicate/ provide education to others** | **Training in how to communicate/ provide education to others** |

| | | | |
|---|---|---|---|
| **Teach Skills** | | Advocacy meetings with members of a church or CBO to teach the importance of vaccination and how to communicate the message | Campaign training day where vaccinators are taught how to communicate with parents<br><br>Training day for vaccinators before the launch of the rotavirus vaccine |
| | **Parenting skills programs** | | |
| **Provide Support** | **One on one interactions** | | |
| | **Group interactions** | | |
| | **Phone-based** | | |
| | **Web-based** | | |
| **Facilitate Decision Making** | **Decision aids** | | **Decision aids** |
| **Enable Communication** | **Interpreters** | | |
| **Enhance Community Ownership** | **Program delivery**<br>Health workers who encourage users to talk about EPI or the campaign in the neighbourhoods | **Local opinion leaders**<br>The quarter head who is mobilized to gather people on vaccination day | |
| | | **Community input**<br>Members of the community (including children) who help vaccination teams to find non-vaccinated children | |
| | | **Program delivery** | |
| | | **Community coalition**<br>Meetings to support groups in the community who work with vaccination such as churches, women's groups and health committees<br><br>**Partnership building**<br>Partnerships with local CBOs, churches and mosques<br><br>Social Mobilizers who are selected by their communities | |
